# Short-Term Particulate Air Pollution Exposure is Associated with Increased Severity of Respiratory and Quality of Life Symptoms in Patients with Fibrotic Sarcoidosis

**DOI:** 10.3390/ijerph15061077

**Published:** 2018-05-26

**Authors:** Cheryl S. Pirozzi, Daniel L. Mendoza, Yizhe Xu, Yue Zhang, Mary Beth Scholand, Robert P. Baughman

**Affiliations:** 1 Division of Pulmonary and Critical Care Medicine, Department of Internal Medicine, University of Utah, Salt Lake City, UT 84132, USA; Daniel.Mendoza@utah.edu (D.L.M.); mary.beth.scholand@hsc.utah.edu (M.B.S.); 2 Department of Atmospheric Sciences, University of Utah, Salt Lake City, UT 84112, USA; 3 Department of Population Health Science, University of Utah, Salt Lake City, UT 84132, USA; yizhe.xu@hsc.utah.edu; 4 Divisions of Epidemiology and Public Health, Department of Internal Medicine, University of Utah, Salt Lake City, UT 84132, USA; zhang.yue@hsc.utah.edu; 5 Department of Internal Medicine, University of Cincinnati, Cincinnati, OH 45219, USA; BAUGHMRP@ucmail.uc.edu

**Keywords:** sarcoidosis, air pollution, particulate matter, ozone, signs and symptoms, respiratory, pulmonary function tests

## Abstract

This study aimed to determine if short-term exposure to particulate matter (PM_2.5_) and ozone (O_3_) is associated with increased symptoms or lung function decline in fibrotic sarcoidosis. Sixteen patients with fibrotic sarcoidosis complicated by frequent exacerbations completed pulmonary function testing and questionnaires every three months for one year. We compared 7-, 10-, and 14-day average levels of PM_2.5_ and O_3_ estimated at patient residences to spirometry (forced expiratory volume in 1 s (FEV1), to forced vital capacity (FVC), episodes of FEV1 decline > 10%) and questionnaire outcomes (Leicester cough questionnaire (LCQ), Saint George Respiratory Questionnaire (SGRQ), and King’s Sarcoidosis Questionnaire (KSQ)) using generalized linear mixed effect models. PM_2.5_ level averaged over 14 days was associated with lower KSQ general health status (score change −6.60 per interquartile range (IQR) PM_2.5_ increase). PM_2.5_ level averaged over 10 and 14 days was associated with lower KSQ lung specific health status (score change −6.93 and −6.91, respectively). PM_2.5_ levels were not associated with FEV_1_, FVC, episodes of FEV_1_ decline > 10%, or respiratory symptoms measured by SGRQ or LCQ. Ozone exposure was not associated with any health outcomes. In this small cohort of patients with fibrotic sarcoidosis, PM_2.5_ exposure was associated with increased severity of respiratory and quality of life symptoms.

## 1. Introduction 

Chronic sarcoidosis progresses to fibrotic disease in approximately 10–20% of patients [1]. Fibrotic disease is characterized by a progression of chronic inflammation to fibrotic transformation, and may include upper and middle lung predominant findings of traction bronchiectasis, bronchial distortion, linear opacities, fibrotic masses, honeycombing, or cysts [1,2]. 

Patients with fibrotic sarcoidosis commonly experience acute episodes of clinical worsening and lung function decline [3,4]. These acute pulmonary exacerbations of sarcoidosis (APES) have been described by decline in pulmonary function, worsening pulmonary symptoms such as cough, sputum, and shortness of breath, increase in biomarkers of disease activity, need to start or restart corticosteroid therapy, and exclusion of alternative causes of pulmonary symptoms and dysfunction [3,4,5]. Risk factors for exacerbations include underlying bronchiectasis, longer disease duration, African American race, previous steroid treatment, and anti-tumor necrosis factor (TNF) therapy [4]. Acute pulmonary exacerbations are typically treated with antibiotics and/or corticosteroid therapy [3,4,5].

Short-term air pollution exposure is associated with exacerbations of other pulmonary diseases such as chronic obstructive pulmonary disease (COPD) [6], asthma [7], and cystic fibrosis [8], which share clinical characteristics with exacerbations of fibrotic sarcoidosis. The effect of elevated short-term air pollution exposure on sarcoidosis is unknown. We aimed to evaluate if short-term exposure to ozone and fine particulate matter (PM_2.5_) is associated with increased symptoms or lung function decline in an exploratory study of a small cohort of patients with fibrotic sarcoidosis.

## 2. Materials and Methods

### 2.1. Study Participants

We performed a secondary analysis of an electronic database of adult fibrotic sarcoidosis cases generated for a separate study [9]. All patients in this analysis had testing performed at the University of Cincinnati Medical Center. Patients with a history of two or more acute exacerbations in the previous year were enrolled in a randomized controlled trial evaluating roflumilast for reducing episodes of acute exacerbation in fibrotic sarcoidosis. All participants had experienced at least two exacerbations of their sarcoidosis in the prior year, with an exacerbation defined as an acute event requiring increase of prednisone, with or without use of antibiotics, and no other etiology identified [4]. For inclusion in the study, patients were between 18 and 70 years of age and had a diagnosis of sarcoidosis by American Thoracic Society (ATS) criteria [10], ratio of forced expiratory volume in 1 s (FEV_1_) to forced vital capacity (FVC) of less than 80%, fibrosis on chest X-ray and/or high-resolution computed tomography (CT) of the chest, and were on a stable dose of corticosteroids and other agents for sarcoidosis for at least four weeks. Exclusion criteria included renal dysfunction with creatinine > 3 mg/dL, moderate or severe liver disease, unstable cardiac disease, non-cutaneous malignancy treated in the past two years, unable to complete questionnaires or lung function testing. Participants completed testing every three months for one year, including spirometry, 6-min walk test, blood draw, and questionnaires. Pulmonary function testing was performed and interpreted according to the 2005 ATS and European Respiratory Society (ERS) guidelines [11,12], and pre-bronchodilator spirometric measurements were used for analysis. For this analysis, we included all patients who completed three or more testing visits. We then excluded two individuals residing in areas lacking air quality data.

### 2.2. Exposure Estimation

Eighteen air quality monitors in southwest Ohio were used for exposure estimation in this study; six recorded fine particulate matter (PM_2.5_) data only, eight recorded ozone data only, and four recorded both PM_2.5_ and ozone data. PM_2.5_ data was available at least every three days (one site provided daily data). Daily ozone data was available between the months of April and October (three monitors provided year-round data) during the study period (2013–2015). The data was gap-filled using linear interpolation to obtain daily values for each pollutant. The home street addresses for 16 patients (14 in the Cincinnati, OH, metro area, 1 in Dayton, OH, and 1 in Logan, OH, approximately 50 miles southeast of Columbus, OH) were geocoded to latitude/longitude coordinates. Using the home locations, daily pollutant exposure was estimated by kriging using the spherical semivariogram model [13]. Average pollutant exposure for 7, 10, and 14 days preceding each study visit was calculated for each patient. The interquartile range (IQR) was calculated for each pollutant and time period combination.

### 2.3. Outcomes

We evaluated three lung function outcomes and four questionnaire outcomes. Lung function outcomes included pre-bronchodilator FEV_1_ and FVC measured in liters, and episodes of FEV_1_ decline greater than 10% from each individual’s highest value. Lung function testing used Snowbird Criteria and Hankinson reference values and lung volumes were corrected for race [12,14].

Questionnaire outcomes included scores from Leicester cough questionnaire (LCQ), Saint George Respiratory Questionnaire (SGRQ), and King’s Sarcoidosis Questionnaire (KSQ) general health status and lung specific health status. Leicester cough questionnaire (LCQ) is a 19 item quality of life measure of cough over the prior two weeks [15]. Saint George Respiratory Questionnaire (SGRQ) measures the impact of respiratory symptoms on overall health, daily life, and perceived well-being, and queries symptoms over the past three months and “these days” [16]. King’s Sarcoidosis Questionnaire is a validated questionnaire for assessing health status in sarcoidosis and consists of five modules (General health status, Lung, Skin, Eye, Medications) [17]. The general health status collects information regarding ten general health and quality of life symptoms occurring over the past two weeks: frustration, trouble concentrating, lack of motivation, tiredness, anxiousness, muscle and joint pains, embarrassment, worry about weight, worry about sarcoidosis, and tiredness interfering with normal social activities. The lung specific health status collects information regarding six respiratory symptoms occurring over the past two weeks: cough causing pain/discomfort; breathlessness climbing stairs or walking up slight inclines; having to take deep breaths, also known as “air hunger”; chest feeling tight; episodes of breathlessness; and chest pains.

### 2.4. Statistical Analysis

We employed generalized linear mixed effect models to evaluate the adjusted association between average PM_2.5_ and ozone exposure and lung function and questionnaire outcomes. All models adjusted for age, gender, smoking status, and study drug assignment. We estimated the coefficients and adjusted odds ratio (aOR) for each outcome associated with an IQR increase in average PM_2.5_ or ozone level. Statistical analyses were conducted using R [18]. 

## 3. Results

Sixteen patients living in Ohio completed three or more testing visits between June 2013 and June 2015 and had complete pollutant exposure data available for their home address. There were a total of 69 testing visits, with an average of 3.6 per patient.

Patient characteristics and outcomes are shown in Table 1.

We observed low to moderate levels of PM_2.5_ and ozone during the study period, shown in Table 2. PM_2.5_ levels were similar between warm and cold seasons, with a mean 14-day average level of 11.9 μg/m^3^ and maximum of 23.5 μg/m^3^. Ozone levels were higher during the warm season, with mean 46 ppb and maximum 55 ppb. Interquartile range (IQR) for PM_2.5_ averaged over 7, 10, and 14 days was 4.92, 4.64, and 4.38 μg/m^3^, respectively. IQR for ozone averaged over 7, 10, and 14 days was 0.014, 0.014, and 0.014 ppm, respectively.

**Figure 1:**
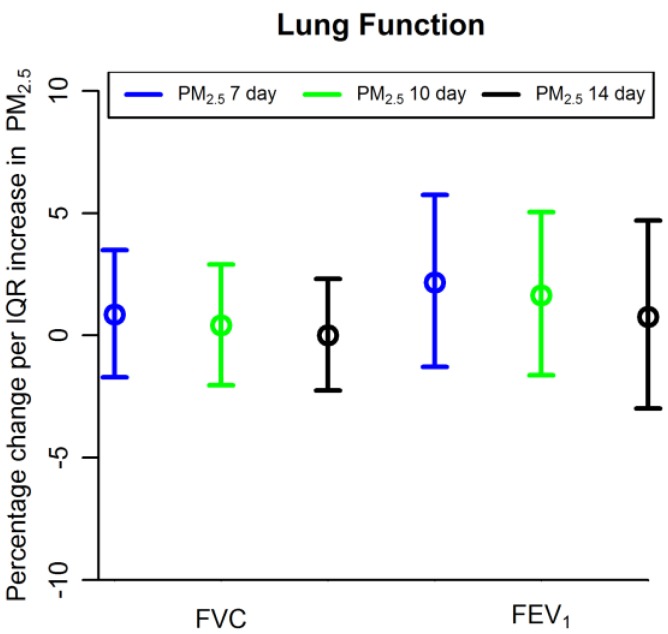
Association between particulate matter (PM_2.5_) and lung function outcomes. Shown are percentage change of FEV_1_ and FVC per interquartile range (IQR) increase in PM_2.5_ averaged over 7, 10, and 14 days. FEV_1_: forced expiratory volume in 1 s. FVC: forced vital capacity.

Table 3 and Figure 1 show results for lung function outcomes. Average PM_2.5_ was not associated with FVC (percentage change <0.01 per IQR increase in 14-day average PM_2.5_, 95% confidence interval (CI) −2.25 to 2.31), FEV_1_ (percentage change 0.76 per IQR increase in 14-day average PM_2.5_, CI −2.99 to 4.70), or episodes of FEV_1_ decline > 10% (Odds ratio (OR) 0.85 per IQR increase in 14-day average PM_2.5_, CI 0.32 to 1.38). Average ozone was not associated with FVC (percentage change 0.80 per IQR increase in 14-day average ozone, CI −3.13 to 4.88), FEV_1_ (percentage change −0.03, CI −5.18 to 5.40), or episodes of FEV_1_ decline > 10% (OR 0.98, CI 0.04 to 1.93).

**Figure 2:**
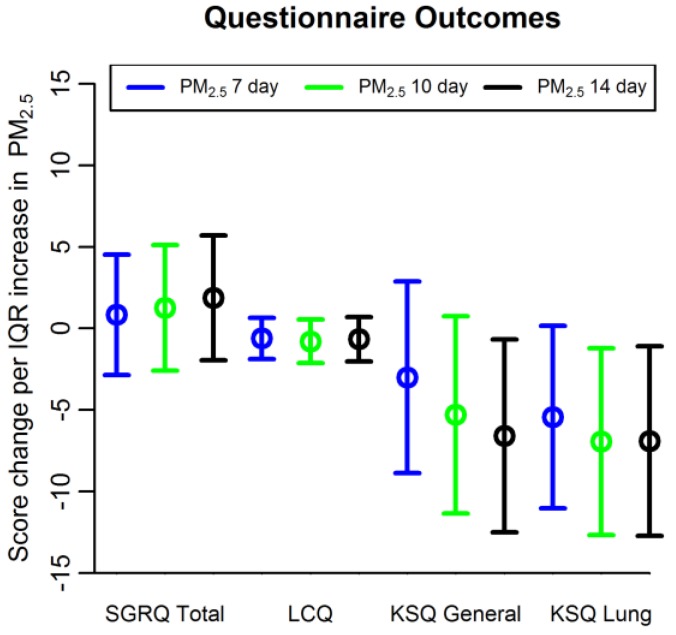
Association between PM_2.5_ and questionnaire outcomes. Shown are questionnaire score changes per interquartile range (IQR) increase in PM_2.5_ averaged over 7, 10, and 14 days. SGRQ = St. George’s Respiratory Questionnaire; LCQ = Leicester Cough Questionnaire; KSQ = King’s Sarcoidosis Questionnaire.

Table 4 and Figure 2 show results for questionnaire outcomes. PM_2.5_ level averaged over 14 days was associated with lower KSQ general health status (score change −6.60 per IQR increase in 14-day average PM_2.5_, 95% confidence interval (CI) −12.51 to −0.68). PM_2.5_ level averaged over 10 and 14 days was associated with lower KSQ lung specific health status (score change −6.93 per IQR increase in 10-day average PM_2.5_, 95% confidence interval (CI) −12.67 to −1.21, and score change −6.91 per IQR increase in 14-day average PM_2.5_, CI −12.73 to −1.09). PM_2.5_ levels were not associated with respiratory symptoms measured by SGRQ (score change 1.87 per IQR increase in 14-day average PM_2.5_, CI −1.96 to 5.70) or LCQ (score change −0.66, CI −2.03 to 0.70). Short-term ozone exposure was not associated with respiratory symptoms measured by SGRQ, LCG, or KSQ. 

## 4. Discussion

In this small cohort of patients with fibrotic sarcoidosis, increased PM_2.5_ exposure was associated with increased severity of respiratory and quality of life symptoms indicated by a decrease in lung specific health status and general health status using the validated King’s Sarcoidosis Questionnaire. These findings suggest that short-term exposure to PM_2.5_ may adversely affect patients with fibrotic sarcoidosis.

To our knowledge, this is the first study to evaluate effects of short-term air pollution exposure on health outcomes in fibrotic sarcoidosis. Prior epidemiologic studies have demonstrated associations between environmental exposures and development of sarcoidosis. These have included microbially-rich environments such as agricultural exposures and mold and mildew, insecticides, industrial organic and inorganic dust, particulate matter and debris from the World Trade Center disaster, and metal industries [19,20,21,22,23,24,25]. These studies suggest that exposure to many different antigens in the environment and workplace may play a role in triggering a granulomatous immune response leading to sarcoidosis. Our study suggests that environmental exposures may also contribute to episodes of clinical worsening in this disease.

We did not detect an association of air pollution exposure with lung function outcomes. Even in the absence of objective changes in pulmonary function, increased respiratory symptoms carry significance for patients’ overall health status. In idiopathic pulmonary fibrosis, dyspnea is a stronger prognostic parameter than other physiologic markers in predicting survival [26]. Patients with pulmonary fibrosis due to sarcoidosis have increased risk for respiratory failure and death and this phase of the disease does not respond as well to the usual treatments in sarcoidosis [27,28]. Identification of environmental exposures that contribute to clinical worsening may lead to opportunities for clinical improvement by reducing exposure.

We did not find any association of ozone exposure with lung function or symptom outcomes. Ozone levels were very low during this study and therefore we cannot exclude the possibility of health effects at higher levels. Additionally, there was less ozone data available during the winter months of the year due to some monitors only recording during warm months.

There are several important features of our study. We evaluated a well-defined cohort of patients with uncommon and severe manifestations of sarcoidosis. All participants had experienced two or more exacerbations of sarcoidosis requiring pharmacologic treatment in the year prior to the study period. This is the first time air pollution exposure effects have been evaluated in this specific disease state. Multiple evaluations of lung function and respiratory symptoms allowed for an evaluation of exposures over multiple time points. We were able to estimate air pollutant exposure at individual place of residence based on data from 18 monitors.

We recognize several limitations to our study. Exposure measures were imperfect, as they were based on observations from multiple monitoring stations recording pollutant data only every three days, and some ozone monitors were without year-round measurements. Similarly, the pollutant concentration estimates were made only for the place of residence, ignoring variability in exposure due to time spent indoors and at locations other than the primary residence. We were only able to evaluate effects of PM_2.5_ and ozone due to a limited number of monitoring stations in the region measuring other air pollutants. Air pollutants other than PM_2.5_ and ozone may have also contributed to respiratory symptoms. PM_2.5_ and ozone exposure levels in this region of Southwest Ohio, USA, were low to moderate, and thus our findings may not accurately reflect exposure effects for patients residing in regions with more extreme exposure patterns. Our sample size was very small, thus we were unable to accurately estimate the effect size, and can only identify that there is an association between PM_2.5_ exposure and increased respiratory symptoms. We consider this study to be exploratory, and our findings suggest further study with larger sample sizes is warranted to better explore the association of short-term air pollution exposure with health outcomes in fibrotic sarcoidosis.

## 5. Conclusions

In this exploratory study evaluating a small cohort of patients with fibrotic sarcoidosis, increased PM_2.5_ exposure was associated with increased severity of respiratory and quality of life symptoms indicated by a decrease in lung-specific and general health status using the validated King’s Sarcoidosis Questionnaire. A larger sample size is needed to evaluate the association of short-term air pollution exposure with other health outcomes in fibrotic sarcoidosis.

## Figures and Tables

**Table 1:** Patient characteristics and outcomes.

Characteristic	Statistic
Age, median years (IQR)	59 (53.25, 62.5)
Female *N* (%)	12 (75%)
African American *N* (%)	9 (56%)
Current smoker *N* (%)	1 (6%)
Former smoker *N* (%)	10 (62%)
Assigned study drug	8 (50%)
FEV1 (L) (mean (SD))	1.61 (0.68)
FEV1 % predicted (mean (SD))	62.38 (21.45)
FVC (L) (mean (SD))	2.32 (0.86)
FVC % predicted (mean (SD))	69.56 (19.81)
Had episode of FEV1 drop > 10% *N* (%)	5 (31%)

Patient characteristics and outcomes of study participants (*N* = 16). IQR: interquartile range; FEV1: forced expiratory volume in 1 s; FVC: forced vital capacity.

**Table 2:** PM_2.5_ and O_3_ levels during the study period.

Pollutant	Time Period	Statistic	7-Day Average	10-Day Average	14-Day Average
**PM_2.5_ (μg/m^3^)**	All days (*N* = 69)	Mean (SD)	11.6 (4.5)	11.9 (4.3)	11.9 (3.8)
		Median (IQR)	10.6 (4.9)	10.9 (4.6)	11.2 (4.4)
		Range	(5.8, 25.3)	(6, 24.7)	(6, 23.5)
	May–October (*N* = 33)	Mean (SD)	10.7 (3.1)	11.3 (2.8)	11.6 (2.7)
		Median (IQR)	10.7 (3.0)	11.2 (3.4)	11.4 (4.5)
		Range	(6.1, 19.1)	(6.1, 17.8)	(6, 17)
	November–April (*N* = 36)	Mean (SD)	12.5 (5.5)	12.5 (5.4)	12.1 (4.6)
		Median (IQR)	10.6 (6.7)	10.2 (6.1)	10.3 (5.4)
		Range	(5.8, 25.3)	(6, 24.7)	(6.1, 23.5)
**O_3_ (ppm)**	All days (*N* = 69)	Mean (SD)	0.04 (0.009)	0.041 (0.008)	0.041 (0.009)
		Median (IQR)	0.041 (0.014)	0.042 (0.014)	0.043 (0.014)
		Range	(0.023, 0.058)	(0.023, 0.056)	(0.023, 0.055)
	May–October (*N* = 33)	Mean (SD)	0.044 (0.007)	0.045 (0.007)	0.046 (0.006)
		Median (IQR)	0.044 (0.008)	0.047 (0.008)	0.048 (0.007)
		Range	(0.026, 0.058)	(0.027, 0.056)	(0.032, 0.055)
	November–April (*N* = 36)	Mean (SD)	0.037 (0.008)	0.037 (0.008)	0.037 (0.008)
		Median (IQR)	0.037 (0.012)	0.036 (0.012)	0.035 (0.013)
		Range	(0.023, 0.052)	(0.023, 0.052)	(0.023, 0.052)

7-, 10-, and 14-day average levels of PM_2.5_ and O_3_ occurring during the study period.

**Table 3:** Association between air pollution exposure and lung function outcomes.

Lung Function Outcome	PM_2.5_			Ozone		
	7-Day Average	10-Day Average	14-Day Average	7-Day Average	10-Day Average	14-Day Average
FVC (L) (% change) ^1^	0.86 (−1.71, 3.50)	0.41 (−2.03, 2.91)	0.0 (−2.25, 2.31)	0.59 (−3.51, 4.86)	0.46 (−3.58, 4.66)	0.80 (−3.13, 4.88)
FEV_1_ (L) (% change) ^1^	2.17 (−1.28, 5.75)	1.65 (−1.63, 5.04)	0.76 (−2.99, 4.70)	0.57 (−4.83, 6.29)	1.11 (−4.25, 6.77)	−0.03 (−5.18, 5.40)
Episodes of FEV_1_ > 10% decline ^2^	0.74 (0.18, 1.19)	0.76 (0.30, 1.22)	0.85 (0.32, 1.38)	0.9 (0.02, 1.79)	0.93 (0.01, 1.85)	0.98 (0.04, 1.93)

Adjusted association between short-term air pollution exposure (PM_2.5_ and ozone) and lung function outcomes in fibrotic sarcoidosis. Shown are ^1^ percentage change (95% confidence interval) of FEV_1_ and FVC for each IQR change in air pollution exposure, and ^2^ odds ratios (95% confidence interval) for episodes of FEV_1_ decline > 10% for each IQR change in air pollution exposure. Interquartile range (IQR) for PM_2.5_ averaged over 7, 10, and 14 days = 4.92, 4.64, and 4.38 μg/m^3^, respectively. IQR for ozone averaged over 7, 10, and 14 days = 0.014, 0.014, and 0.014 ppm, respectively. All models adjusted for age, sex, smoking status, and study drug assignment. PM_2.5_ = fine particulate matter with diameter less than 2.5 μm; FEV_1_: forced expiratory volume in 1 s; FVC: forced vital capacity.

**Table 4:** Association between air pollution exposure and questionnaire outcomes.

Questionnaire Outcome	PM_2.5_			Ozone		
	7-Day Average	10-Day Average	14-Day Average	7-Day Average	10-Day Average	14-Day Average
SGRQ Total Score	0.84 (−2.85, 4.53)	1.26 (−2.59, 5.11)	1.87 (−1.96, 5.70)	−0.33 (−4.73, 4.07)	−1.08 (−5.5, 3.3)	−0.87 (−5.41, 3.66)
LCQ score	−0.61 (−1.87, 0.65)	−0.80 (−2.13, 0.54)	−0.66 (−2.03, 0.70)	−1.00 (−2.57, 0.57)	−0.97 (−2.55, 0.61)	−0.84 (−2.45, 0.77)
KSQ General Health Status	−3.00 (−8.87, 2.88)	−5.3 (−11.35, 0.75)	−6.60 (−12.51, −0.68) *	−1.22 (−8.88, 6.44)	−0.69 (−8.5, 7.1)	−0.48 (−8.37, 7.42)
KSQ Lung Health Status	−5.44 (−11.03, 0.15)	−6.93 (−12.67, −1.21) *	−6.91 (−12.73, −1.09) *	−3.57 (−10.9, 3.75)	−2.85 (−10.28, 4.58)	−2.34 (−9.98, 5.19)

Adjusted association between short-term air pollution exposure (PM_2.5_ and ozone) and questionnaire outcomes in fibrotic sarcoidosis. Shown are regression coefficients (95% confidence interval) indicating questionnaire score change per IQR increase in air pollution exposure, * = *p* value < 0.05 for association. Interquartile range (IQR) for PM_2.5_ averaged over 7, 10, and 14 days = 4.92, 4.64, and 4.38 μg/m^3^, respectively. IQR for ozone averaged over 7, 10, and 14 days = 0.014, 0.014, and 0.014 ppm, respectively. All models adjusted for age, sex, smoking status, and study drug assignment. SGRQ: St. George’s Respiratory Questionnaire; LCQ: Leicester Cough Questionnaire; KSQ: King’s Sarcoidosis Questionnaire.

## List of Abbreviations

O_3_	ozone
PM_2.5_	fine particulate matter with diameter less than 2.5 μm
FEV_1_	forced expiratory volume in 1 s
FVC	forced vital capacity
SGRQ	St. George’s Respiratory Questionnaire
LCQ	Leicester Cough Questionnaire
KSQ	King’s Sarcoidosis Questionnaire
IQR	interquartile range
APES	acute pulmonary exacerbations of sarcoidosis
